# Diagnosis of Nephropathic Cystinosis in a Child During Routine Eye Exam

**DOI:** 10.4274/tjo.69922

**Published:** 2017-10-27

**Authors:** Mahmut Ecel, Ayça Sarı, Ali Delibaş

**Affiliations:** 1 Private Tarsus Medical Park Hospital, Ophthalmology Clinic, Mersin, Turkey; 2 Mersin University Faculty of Medicine, Department of Ophthalmology, Mersin, Turkey; 3 Mersin University Faculty of Medicine, Department of Pediatric Nephrology, Mersin, Turkey

**Keywords:** Cystinosis, Cornea, nephropathy

## Abstract

We present a 7-year-old patient who was diagnosed with asymptomatic nephropathic cystinosis following the detection of the pathognomonic corneal white crystalline opacities during a routine eye examination.

## INTRODUCTION

Cystinosis is a rare metabolic disease with autosomal recessive inheritance,^1^ characterized by the accumulation of cystine crystals in various tissues including the kidneys, bone marrow, pancreas, thyroid, muscle, brain, and eyes.^2,3^ Cystinosis can clinically manifest as a nephropathic or non-nephropathic form. The nephropathic form is further divided into two subtypes: infantile cystinosis and juvenile cystinosis.^4^ The non-nephropathic form is also known as ocular cystinosis. In ocular cystinosis, pathognomonic yellow-white crystalline deposits are observed in the cornea, but the kidneys are not affected.^4^

## CASE REPORT

A 7-year-old female patient with no complaints presented to our clinic for routine eye examination. Her uncorrected vision was perfect in both eyes. On anterior segment examination, yellowish-white crystallized opacities were observed throughout the corneal stroma bilaterally (Figure 1A, 1B). Anterior segment examination was otherwise unremarkable and fundus examination was normal in both eyes. The patient was referred to the university hospital with a prediagnosis of cystinosis. In the biochemical analyses done at the hospital, urinalysis revealed hemoglobin 1+ and protein 2+; 24-hr urine analysis results were phosphorus: 14.4 mg/dL (phosphaturia), creatinine: 16.6 mg/dL (reduced clearance), phosphorus: 3.73 mg/dL (hypophosphatemia); and blood biochemistry test showed normal albumin, calcium, and sodium levels. The patient was diagnosed with nephropathic cystinosis based on these findings and the presence of ocular crystalline deposits. Following cysteamine treatment, clearance and phosphorus levels returned to normal in the follow-up 24-hr urinalysis. Treatment with topical 0.05% cysteamine drops 5 times daily was initiated for the corneal opacities. At 1-year follow-up, the patient had perfect vision in both eyes. Although the topical cysteamine therapy had not reduced the opacities in the cornea caused by cystine crystals, no ocular or systemic complications were observed.

## DISCUSSION

Cystinosis is a lysosomal storage disease with an autosomal recessive inheritance pattern. Impairment of the transporter system responsible for transporting cystine out of lysosomes results in the accumulation of cystine crystals in tissues such as the kidneys, eyes, bone marrow, liver, spleen, pancreas, thyroid, skeletal muscles, thyroid, and brain.^4^ Electron microscopic and ultrastructural examinations of the crystals have shown that they formed of intralysosomal L-cysteine.^5^ Most of the cases in the literature are of infantile type, which is the clinically most severe form.^6^

Ocular cystinosis is diagnosed when the pathognomonic refractive cystine crystal accumulations are observed throughout the conjunctiva and entire cornea (central and peripheral) on anterior segment examination.^6^ These deposits can be located in the corneal epithelium, stroma, and endothelium, and their distribution may be associated with disease course and duration.^7^ The corneal crystals begin to accumulate in infancy, being found in almost all cases of nephropathic cystinosis at 16 months.^7^ They first accumulate in the peripheral cornea and progress toward the center with age.^8^ While these crystalline deposits often do not cause visual impairment, in rare cases they may form band keratopathy, thus affecting the central cornea and reducing visual acuity.^9^ Superficial punctate and filamentary keratopathy are frequently observed in adults, whereas band keratopathy, peripheral corneal neovascularization, and posterior synechiae associated with increased iris thickness are seen in the elderly.^10^ Rare long-term complications of infantile and juvenile cystinosis include patchy pigmentary retinopathy, pigmentary changes in the macula, and involvement of the iris, ciliary body, choroid, and optic nerve due to the cystine accumulations.^10^ The most common symptoms in older patients is photophobia and associated blepharospasm.^11^ In addition, color vision, peripheral vision, and night vision may be reduced due to anterior and posterior segment complications.^11^ Depigmentation of the peripheral retina with pigment epithelium mottling is the most common posterior segment complication.^12^ In about 10-15% of patients, retinopathy leads to blindness.^13^

Ocular cystinosis is considered one of the corneal crystalline keratopathies. The term crystalline keratopathy describes a group of diseases in which crystal deposits form on the anterior surface of the corneal epithelium or stroma due to a variety of reasons such as infection, corneal dystrophies, or systemic causes. The etiology of crystalline keratopathy may be ocular, systemic, or medication-induced. Infectious causes include *de novo*, recent refractive or corneal surgeries, and interventions such as keratoplasty. Corneal dystrophies that cause crystalline keratopathy include Schnyder crystalline cornea dystrophy and Bietti crystalline corneoretinal dystrophy. Schnyder dystrophy is a slowly progressive corneal dystrophy with autosomal dominant inheritance.^14^ Clinical manifestations include opacification of the central or midperipheral cornea, dense arcus senilis, reduced corneal sensitivity, and recurrent corneal erosion.^15^ Bietti corneal dystrophy shows an autosomal recessive inheritance pattern and is characterized by progressive night blindness and narrowing of the visual field. Clinical manifestations include sparkling, yellowish retinal crystals resembling chalk powder, choroidal atrophy and sclerosis, and yellow-white crystals in the superficial stroma and subepithelial layer of the peripheral cornea. Monoclonal gammopathy and multiple myeloma lymphoproliferative disorders may also cause crystalline keratopathy.^16^ Corneal accumulations may also form in the epithelium or stroma. Diagnosis is made by conjunctival biopsy, or blood or bone marrow smear. Drug-induced crystalline keratopathy may also occur with the use of fluoroquinolone (ciprofloxacin) drops. The effects usually resolve spontaneously when the topical fluoroquinolone is discontinued.

Infantile nephropathic cystinosis accounts for 95% of cystinosis cases and is the most severe form of the disease. The renal phenotype consists of Fanconi syndrome, in which progressive glomerular dysfunction and loss eventually lead to end-stage renal failure.^17^ It initially manifests as asymptomatic aminoaciduria. Proximal tubular dysfunction which develops between 6-12 months of age leads to the loss of amino acids, sodium, potassium, bicarbonate, magnesium, carnitine, calcium, phosphate, glucose, and low-to-middle molecular weight proteins in the urine, resulting in the development of Fanconi syndrome.^18^ Infants may present with lack of appetite, polyuria, polydipsia, severe dehydration and electrolyte imbalance, vomiting, constipation, and sometimes vitamin D-resistant rickets. Biochemical tests may reveal hypokalemia, metabolic acidosis, hypophosphatemia, hypocalcemia, low carnitine level, and hyponatremia. If left untreated, end-stage renal failure develops by the end of the first decade.^19^

Five percent of cystinosis patients are diagnosed with the juvenile form. It appears in late childhood or early adolescence.^20^ This form is less severe than nephropathic cystinosis, with milder disease course and symptoms. Children with juvenile cystinosis do not exhibit significant retardation of growth or development.

In 50-70% of patients, fibrosis resulting from accumulation of cystine crystals in thyroid follicular cells causes primary hypothyroidism in the second decade of life.^21^ Patients exhibit subclinical hypothyroidism characterized by normal T3-T4 levels and elevated TSH.

Endocrine and exocrine pancreatic insufficiency often occurs in patients with cystinosis after renal transplantation.^22^ Around the age of 18, 50% of infantile cystinosis patients develop gradual reduction of insulin secretion and C-peptide production, resulting in glucose intolerance and diabetes.

Primary hypogonadism occurs in 70% of male cystinosis patients.^23^ Azoospermia may cause infertility. Female patients are usually asymptomatic.

Central nervous system involvement may cause hypotonia, tremors, delayed speech, gross and fine motor impairment, idiopathic intracranial hypertension, neurocognitive dysfunction, behavioral disorders, and encephalopathy.^23^ Cerebral cortical atrophy, hydrocephalus, demyelination, and vacuolar necrotic brain changes may develop at later ages.

The cysteamine used in the treatment of cystinosis acts by disrupting the disulfide bonds of the cystine molecule, allowing the resulting intermediary metabolites to be eliminated from the body without accumulating. Topical cysteamine therapy administered in ocular cystinosis aims to reduce the cystine crystals that have accumulated in the cornea and to prevent complications they may cause. It has been shown that treating patients for 6 months with topical 0.05% cysteamine drops administered 5 times daily resulted in reduction of corneal cystine crystals, improvement in visual acuity, and decreased photophobia and blepharospasm. However, there was no decrease in corneal crystals after topical cysteamine use in our patient.

Cystinosis is a metabolic disease that causes the accumulation of cystine crystals throughout the body and most commonly affects the eyes and kidneys. Ophthalmologists play an important mediator role in diagnosing asymptomatic cystinosis patients by anterior segment examination, especially during routine eye examination. Patients diagnosed with cystinosis should be referred to pediatric nephrology for systemic involvement and possible complications. Early initiation of cysteamine therapy is beneficial for preventing the development of late-stage renal failure.

## Figures and Tables

**Figure 1A:**
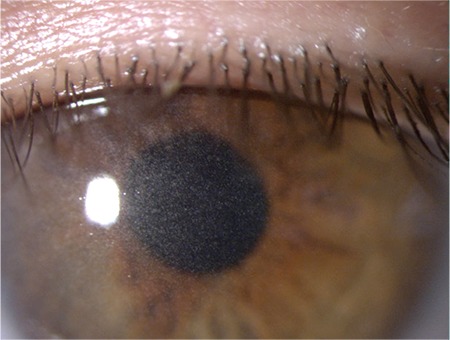
A. Yellowish-white accumulated crystals observed throughout the entire corneal stroma of the right eye

**Figure 1B:**
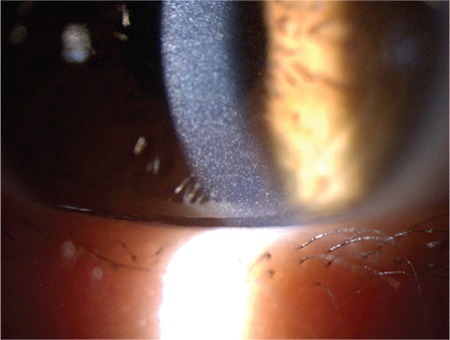
The appearance of cystine crystals in the left eye at a magnification of x16
